# Carotenoid-Enriched Nanoemulsions and γ-Rays Synergistically Induce Cell Death in a Novel Radioresistant Osteosarcoma Cell Line

**DOI:** 10.3390/ijms232415959

**Published:** 2022-12-15

**Authors:** Maria Russo, Stefania Moccia, Carmela Spagnuolo, Idolo Tedesco, Gian Luigi Russo

**Affiliations:** Institute of Food Sciences National Research Council, 83100 Avellino, Italy

**Keywords:** γ-radiation resistance, carotenoids, autophagy, therapy-induced senescence, senolytics

## Abstract

We previously demonstrated that SAOS human osteosarcoma cells, incubated with carotenoid-enriched nanoemulsions (CEN), activated a nonprotective form of autophagy and delayed cell proliferation. The present work focuses on the biological effects of CEN on a derivative of SAOS cells named SAOS400, recently described for their radiation resistance and higher expression of therapy-induced senescence (TIS) markers. SAOS400 cells, incubated with CEN, activated a “cytostatic” form of autophagy confirmed by cell cycle arrest in the G2/M phase and increased expression of autophagic proteins. Treatment of SAOS400 cells with CEN also resulted in decreased expression of the senescence marker p16^INK4^. However, when SAOS400 cells were γ-irradiated in combination with CEN, the threshold for cell death was reached (>60% after 96 h). We showed that this type of cell death corresponded to ‘cytotoxic’ or ‘lethal’ autophagy and that the combined treatment of CEN plus γ-rays was synergistic, with the combination index < 1. Since CEN contained β-carotene, the pure compound was used in SAOS400 cells at the same concentration present in CEN and up to 10 times higher. However, no radio-sensitizing effect of β-carotene was observed, suggesting that the biological effect of CEN was due to less abundant but more bioactive molecules, or to the synergistic activity of multiple components present in the extracts, confirming the functional pleiotropy of natural extracts enriched in bioactive molecules.

## 1. Introduction

Carotenoids represent a well-known class of phytochemicals that have been extensively investigated since the middle of the last century for their chemical, biochemical, and biological properties. Among the more than 1100 carotenoids synthesized by photosynthetic organisms and some non-photosynthetic bacteria and fungi, but not animal cells, approximately 50 are present in the human diet and approximately 20 can be detected in human blood and tissues [1]. Molecular structures are characterized by a long system of conjugated double bonds with π-electrons delocalized throughout the polyene chain, which effectively results in the scavenging of reactive oxygen species (ROS). They are divided into two groups depending on the absence or presence of oxygen: carotenes, formed only by hydrogen and carbon (α-carotene, β-carotene, lycopene), and xanthophylls, consisting of chains containing oxygen atoms, such as lutein, zeaxanthin, and canthaxanthin [1]. Anticancer effects of specific carotenoids (e.g., β-carotene, lycopene, and the intracellular metabolite all-trans retinoic acid, ATRA) and xanthophylls (for example, astaxanthin and fucoxanthin) are associated with a variety of molecular mechanisms. They can modulate hormone and growth factor signaling, affect the regulation of cell cycle and cell differentiation, and induce cell death (apoptosis or type I cell death) and autophagy-dependent cell death (ADCD).

Autophagy (macro-autophagy) is a catabolic process in which macromolecules (proteins, lipids, nucleic acids) and damaged organelles (generally defined cargo) are degraded and recycled into lysosomes by autophagosome formation, providing metabolites to maintain cellular homeostasis [2]. Biochemically, autophagy depends on the coordinated activity of Atg proteins that are at the crossroads of important metabolic pathways: amino acid sensing regulated by the mTOR kinase complex, intracellular ATP content controlled by AMPK (AMP-activated kinase), and a stress signaling mechanism by HIF (hypoxia-inducing factor). All these pathways can activate/disable Atg proteins to obtain a homeostatic effect. In other words, the basal state of a cell is strictly dependent on metabolic (such as ATP depletion) or environmental stress (such as chemotherapy or radiation therapy) [3].

Recent articles investigated the modulation of autophagy by carotenoids in normal and cancer cells. The biological effects of lycopene were explored in the treatment of the cutaneous squamous cell carcinoma cell line COLO-16, human epidermal keratinocytes (HEK), and the immortalized human keratinocyte cell line HaCaT. This study found that lycopene inhibited COLO-16 cell proliferation and migration, but not normal keratinocytes. The cellular effects of lycopene were due to a decrease in autophagic flux in COLO-16 cells based on the inhibition of m-TORC1 [4]. Wang et al. studied the chemopreventive effects of lycopene in vivo in chemically induced cutaneous tumor mice and in cellular models. They showed that lycopene attenuated incidence and multiplicity through an autophagic response dependent on p62^SQSTM1^ and activation of the Nrf2-dependent antioxidant response element (ARE). This study provided preclinical evidence on the chemopreventive effects of lycopene on skin tumors and revealed the mechanistic link between lycopene-mediated stimulation of the Nrf2 signaling pathway and degradation mediated by p62^SQSTM1^ of Keap1, the cytoplasmic inhibitor of Nrf2, through the autophagic lysosomal pathway [5]. In summary, these studies indicate that carotenoids can mainly activate protective autophagic processes in cells that are not transformed, differentiated, or cancerogenic. The effect is probably associated with its ability to modulate intracellular redox status, leading to activation of Nfr2 signaling, correlated with the antioxidative response [1]. However, in cancer cells, carotenoids can be beneficial during the early stages of cancerogenesis, or in other terms, when employed as chemopreventive agents [6]. Unlike polyphenols, few studies refer to the chemotherapeutic use of carotenoids (as single molecules or as adjuvants) in chemotherapy and radiation therapy in tumor models. In this context, it is useful to cite a study in which a carotenoid-enriched extract of *Cucurbita moschata* showed an antiproliferative and differentiating effect associated with blocking autophagic flux and activation of p27^KIP1^ and AMPK in colon cancer (Caco-2) and osteosarcoma (SAOS) cell lines [7]. Similar to autophagy, very few papers refer to senescence as a therapeutic target for carotenoids in cancer cells. Senescence or therapy-induced senescence (TIS) is a common outcome in tissues after clinically relevant doses of irradiation (IR). Clinical evidence revealed that IR, failing to bypass the threshold necessary to trigger apoptosis, can favor repopulation of tumor cells, stimulating protective autophagy or TIS in the subgroup of death-resistant cancer cells [8,9,10]. Pioneering studies focused on an alternative way of therapeutic management of senescent-associated or sustained by senescence: targeted killing of senescent cells (or senolysis) of tumor or stromal origin [11]. Following this initial approach, several small molecules have been identified that selectively kill senescent cells [12,13,14]. Senolytic drugs include BH3 mimetics, such as ABT-263/Navitoclax, tyrosine kinase inhibitors such as Desatinib, and natural molecules belonging to the class of flavonoids, including quercetin and fisetin [14]. There are studies suggesting that carotenoids can act as anti-senescence agents. As an example, β-carotene was applied to HaCAT (human immortalized keratinocyte) cells, resulting in reduced expression of metalloproteinase enzymes (MMP-1, MMP-3, and MMP-10), used as markers of the so-called Senescence-Associated Secretory Phenotype (SASP) [15]. In a different paper, higher concentrations of lutein and zeaxanthin in human plasma were associated with a longer leukocyte telomere length [16]. However, the cellular effects of carotenoids on classic senescence markers, such as SA-β-Galactosidase activity or the expression of key regulators of cell cycle arrest, TP53, p21^Cip1^, and p16^INK4^, have not yet been studied [17].

In this paper, we selected the SAOS cell line, being a well-known preclinical model of osteosarcoma, a type of cancer that can metastasize and become resistant to chemo and radiation therapy and that accounts for 56% of bone neoplasms observed in children. In a previous article [18], we showed that SAOS400 cells, derived from SAOS after two cycles of irradiation and four weeks of in vitro selection, were highly resistant to radiation-induced oxidative stress and were enriched in senescent cells with overexpression of p16^INK4^ and lysosomal β-GAL staining markers with respect to parental cells.

In the current paper, we employed a carotenoid-enriched extract (CEN) previously characterized [19] in association with IR to study its ability to sensitize this new radioresistant cell line, SAOS400, derived from SAOS as recently described [18], to apoptosis, autophagy, and senescence.

It is worthwhile to highlight the main novelties of the present work that reside in testing the senolytic activity of carotenoids, a class of phytochemicals less studied compared to phenolic compounds due to their low solubility and stability. To this aim, we employed a chemical characterization formulation enriched in carotenoids to assess its capacity to counteract radiation resistance in osteosarcoma cells differentially mediated by senescence and autophagy processes.

The present work, even if limited by in vitro cellular models, fills a gap of knowledge in the role of carotenoids in tumor radioresistance that could be due to the extreme hydrophobicity of these molecules and their double role as antioxidant or pro-oxidant molecules [1]. In this scenario, nanoemulsions have evolved as carriers that are particularly attractive for hydrophobic drug delivery (e.g., doxorubicin, fenretinide, and cisplatin) [20,21] and represent an optimal tool for the delivery of other classes of natural molecules even more hydrophobic and chemically instable than flavonoids, such as carotenoids or a phytocomplex enriched in carotenoids, as reported in the present article.

## 2. Results

### 2.1. Evaluation of Autophagy Markers in SAOS and SAOS400 Cell Lines

**Figure 1:**
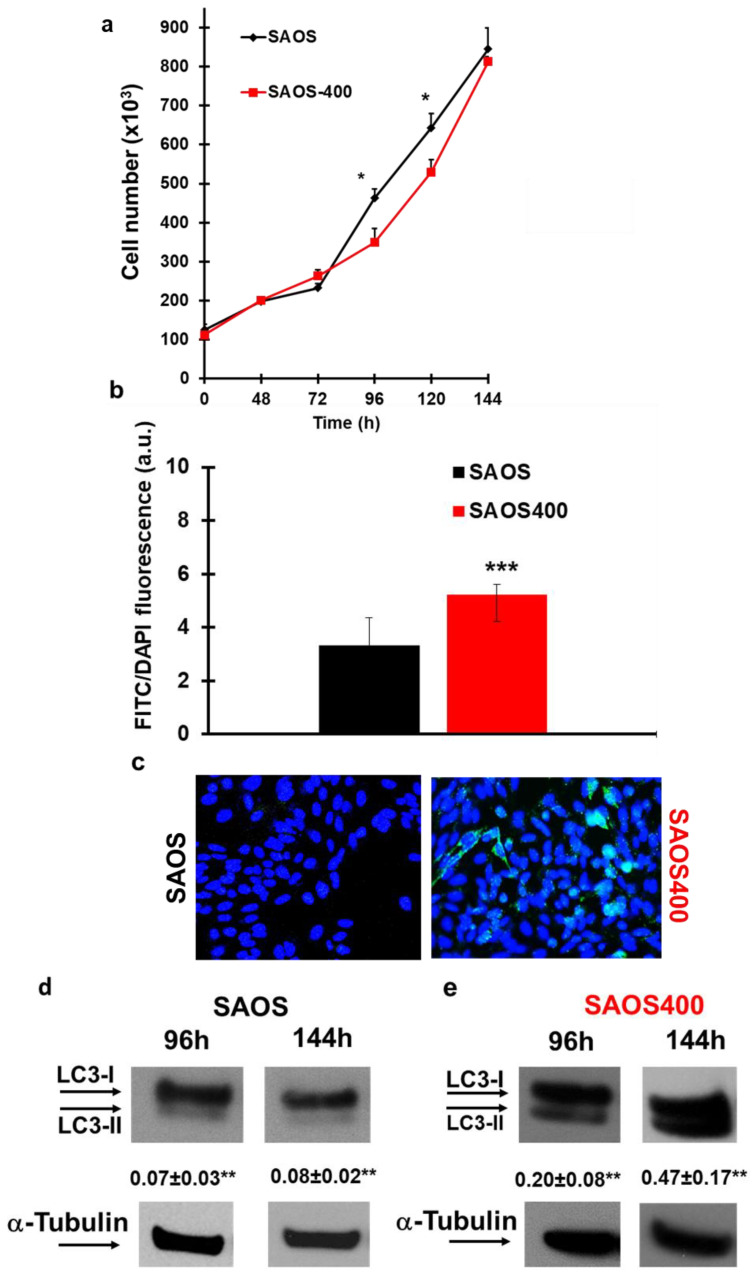
Panel (**a**): Proliferation curve of SAOS- and SAOS400-derived radioresistant cells. Cells were counted in duplicate at the indicated time points with Trypan blue stain using an automatic cell counter, as described. The mean of three experiments is shown ± SD. * *p* < 0.05 SAOS vs. SAOS400, Student’s *t* test. Panels (**b**,**c**): Comparison of the expression of autophagy markers in SAOS vs. SAOS400 cells. Cells were cultured for 96 h after autophagosome staining with Cyto-ID selective dye (FITC). The nuclei were stained with Hoechst dye, and the intracellular number was expressed as the FITC/DAPI ratio. The bar graphs (**b**) represent the mean ± SD, and symbols indicate significance: *** *p* < 0.001, with respect to SAOS. Representative photographs of untreated cells after Cyto-ID staining are reported in (**c**). Panels (**d**,**e**): Immunoblot analysis of the autophagy marker LC3I/II in SAOS and SAOS400 cells cultured at the indicated times. Densitometric analysis (numbers between panels indicate mean ± SD) was calculated by normalizing the expression of LC3-II with α-tubulin, as described in the Materials and Methods section (Section 4). Symbols indicate significance: ** *p* < 0.01 SAOS400 with respect to SAOS cells.

We previously characterized the new SAOS400 cell line in terms of therapy-induced senescence (TIS) as cellular phenotypes associated with radioresistance after genotoxic stress [18]. Here, we deepened our observations on the role of autophagy, specifically therapy-induced autophagy (TIA), by comparing the parental SAOS cell line and its derivative SAOS400. Figure 1a shows that SAOS400 cells slowly duplicated after 96 and 120 h with respect to their parental counterpart. This was not entirely unexpected considering that SAOS400 cells are enriched in senescent cells [18] that usually slow or stop the cell cycle and activate autophagy, trying to repair cell damage. Therefore, we measured intracellular levels of autophagosomes as a marker of the different modulations of autophagic flux. SAOS400 cells showed higher basal levels of intracellular autophagosomes compared to parental cells (>1.6 times) (Figure 1b). This observation was confirmed by elevated levels of lipidated LC3-II at 96 and 120 h (Figure 1d,e), whose increase is always associated with autophagosome formation and regulation of autophagic flux [22].

### 2.2. Effect of CEN on the SAOS400 Cell Line

In a previous work, we demonstrated in SAOS and Caco-2 cells that a carotenoid-enriched extract (CEN) derived from pumpkin *Cucurbita moschata* was able to slow cell proliferation by activating the nonprotective form of autophagy [7]. Here, we evaluated the capacity of CEN to modulate the autophagic process in SAOS400 cells and its ability to bypass radioresistance. Therefore, we tested CEN on SAOS400 cells under the same conditions and concentration (200 μg/mL *w*/*v*) previously applied to SAOS cells [7].

**Figure 2:**
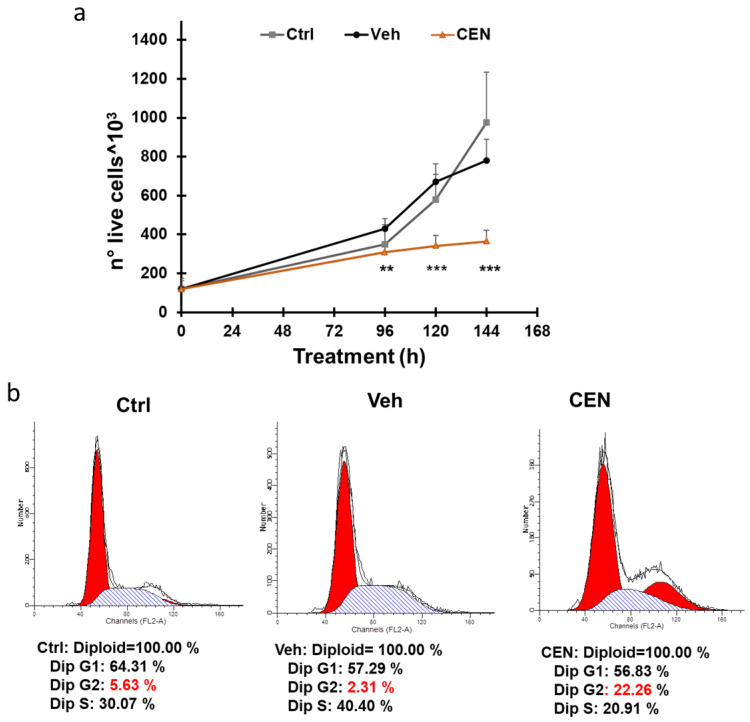
CEN arrested the proliferation of SAOS400 cells in the G2/M phase of the cell cycle. Panel (**a**): Cells were treated with CEN (200 μg/mL) and counted at the indicated times using Trypan blue stain, as described in the Methods Section. The line graphs represent the mean of three experiments (±SD). Symbols indicate significance: ** *p* < 0.01 and *** *p* < 0.001, with respect to untreated (Ctrl) and vehicle (Veh). Panel (**b**): Cells were fixed and stained with propidium iodide for cytofluorimetric analysis, as described. DNA content analyses obtained with ModFit software 3.0 are shown after 144 h of incubation in untreated (Ctrl), vehicle (Veh), or CEN-treated cells.

CEN reduced cell proliferation in SAOS400 cells at different time points (96–144 h; Figure 2a) compared to the vehicle (Veh, empty nanoemulsions) or cell culture medium (Ctrl). Cell count showed the ability of CEN to stop cell proliferation, as confirmed by the cell cycle analysis reported in Figure 2b. Cytofluorimetric analysis indicated that the percentage of SAOS400 cells in phase S decreased by approximately 50%, while the cellular population in phase G2 increased by approximately 10-fold relative to the vehicle. This observation was confirmed after 120 h of CEN treatment, suggesting a *bona fide* capacity of CEN to arrest the cell cycle in the G2/M phase.

### 2.3. CEN Induces Protective Autophagy in the SAOS400 Cell Line

**Figure 3:**
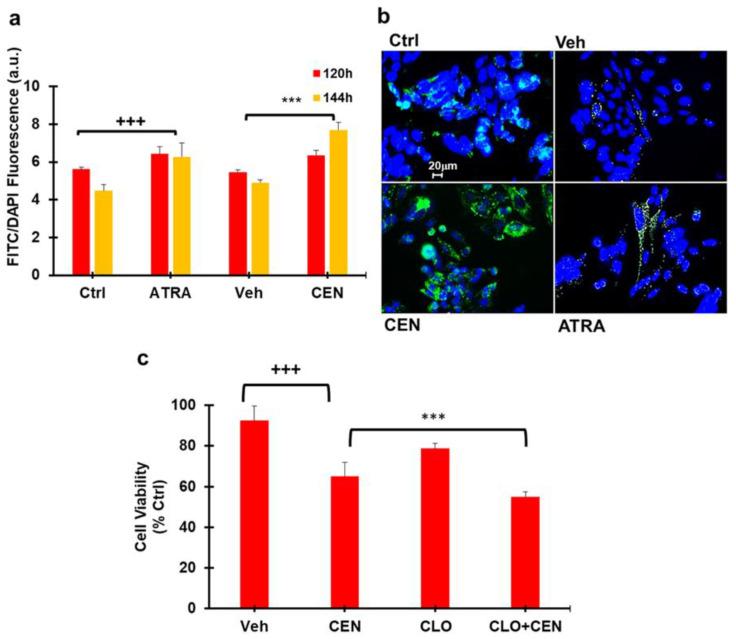
CEN induces protective autophagy in SAOS400 cell lines. Panel (**a**): SAOS400 cells were treated for the indicated time with CEN (200 μg/mL) or ATRA (2 μM) used as a positive control. Cyto-ID staining was used to quantify intracellular autophagosomes and expressed as FITC/DAPI fluorescence, as described in the Materials and Methods Section. Panel (**b**): Micrographs of the representative fields of SAOS400 cells treated as described in (**a**) (400× magnification). Panel (**c**): Cells were treated with CEN (200 μg/mL) or pretreated with chloroquine (CLO; 10 μM) before incubation with CEN for 96 h. The Cristal Violet assay was used to assess cell viability at the end of incubation, as described. The bar graphs represent the mean ± SD, and symbols and linear bars indicate significance: *** *p* < 0.001 of SAOS400 vs. CLO and CEN mono treatments; +++ *p* < 0.001 CEN vs. cells treated with control nanoemulsions (Veh).

To verify whether CEN-induced cell cycle arrest in SAOS400 cells was related to a different modulation of autophagic flux, we used Cyto-ID selective dye to directly quantify intracellular autophagosome after 120 and 144 h of treatment (Figure 3a,b). All-trans retinoic acid (ATRA), which can induce autophagy in SAOS cells, was used as a positive control [23]. Compared to Veh-incubated cells, CEN significantly increased cytoplasmic autophagosome (>16% after 120 h and >56% after 144 h), even to a greater extent than ATRA (>14% after 120 h and >40% after 144 h).

To assess the CEN-induced impact of the autophagic process on cell viability, we used chloroquine (CLO; 10 μM) as a chemical inhibitor of autophagic flux (Figure 3c). In the combined treatment of CLO plus CEN, cell viability was reduced compared to the mono treatments of CEN or CLO (<23% CLO + CEN vs. CLO and <10% CLO + CEN vs. CEN), indicating activation of a protective form of autophagy [24].

### 2.4. CEN Modulates Both Autophagy and Senescence in the SAOS400 Cell Line

**Figure 4:**
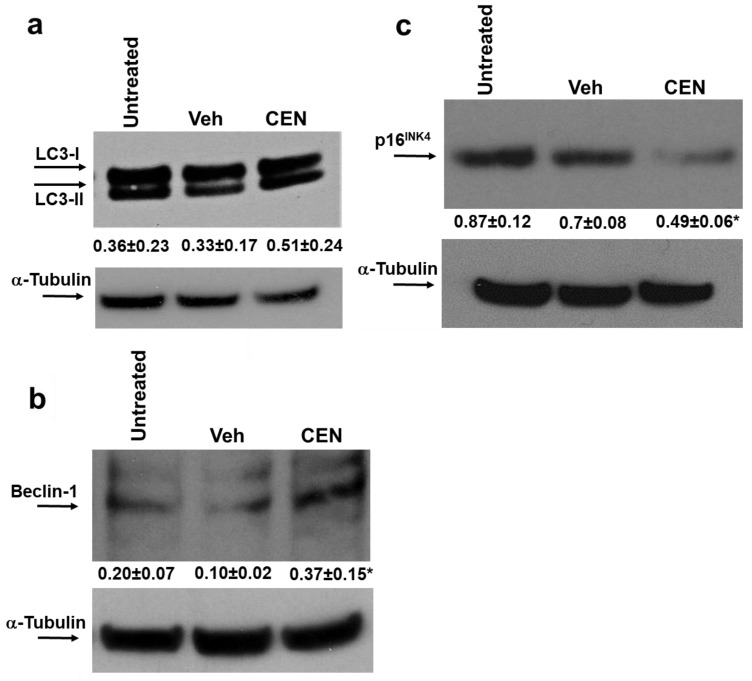
CEN modulates autophagy and senescence in SAOS400 cell lines. The cells were incubated for 120 h with cell culture medium, CEN, or Veh. Cell extracts were immunoblotted to detect autophagy and senescence markers, LC3I/II, Beclin-1 (panel (**a**,**b**)), and p16^INK4^ (panel (**c**)), respectively. Densitometric analysis (numbers between panels indicate mean ± SD) was obtained normalizing the expression of LC3-II, Beclin-1, and p16^INK4^ with the expression of α-tubulin, as described in the Materials and Methods Section. Symbols indicate significance: * *p* < 0.05 CEN with respect to Veh.

To verify how CEN modulated autophagy and senescence, we evaluated the expression of autophagic markers (LC3-I/II and Beclin-1) and senescence markers (p16^INK4^). Figure 4a shows that LC3-II, the lipidated form of LC3, although expressed at high basal levels in SAOS400 cells, was slightly higher after treatment with CEN; however, Beclin-1 was significantly overexpressed after CEN treatment (>3-fold vs. Veh). On the contrary, CEN clearly downregulated p16^INK4^ levels (<2-fold) at the same time of treatment (120 h).

### 2.5. CEN and γ-Rays Do Not Induce Apoptosis and Affect Autophagy and Senescence in the SAOS400 Cell Line

**Figure 5:**
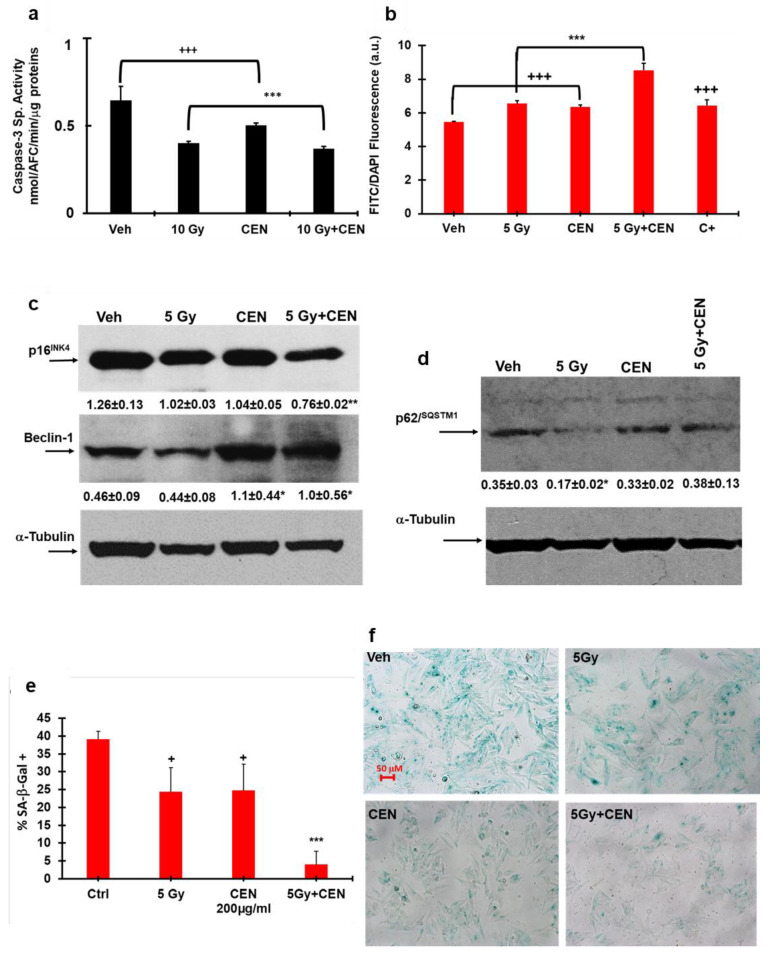
Irradiation and CEN do not induce apoptosis and modulate autophagy and senescence markers differently in SAOS400 cells. Panel (**a**): Caspase-3 enzymatic activity. The cells were irradiated (10 Gy) and then incubated with 200 μg/mL of CEN or their combinations, and after 24 h the cells were harvested, washed in PBS, and resuspended in caspase cell lysis buffer. Caspase-3 activity was measured fluorometrically using the specific substrate DEVD-AFC, as previously described [25]. Bar graphs represent the mean ± SD, and symbols and the linear bar indicate significance: +++ *p* < 0.01 CEN vs. Veh, *** *p* < 0.01 10 Gy and CEN vs. CEN + 10 Gy, Student’s *t* test. (**b**) Cyto-ID staining of SAOS400 cells irradiated with 5 Gy and treated for 120 h with 200 μg/mL of CEN or their combinations. CLO (20 μM) was used as a positive control in this experiment (C+). The bar graphs represent the mean ± SD, and symbols and the linear bar indicate significance: +++ *p* < 0.001 IR and CEN vs. Veh, *** *p* < 0.001 CEN + IR vs. single treatments (Student’s *t* test). Panels (**c**,**d**): Immunoblot analysis of autophagy markers (Beclin-1, p62^SQSTM1^) and senescence markers (p16^INK4^) in SAOS400 cells irradiated with 5 Gy or incubated with 200 μg/mL of CEN and their combination for 144 h. Densitometric analysis (numbers between panels indicate mean ± SD) was calculated normalizing the expression of Beclin-1, p16^INK4^, and p62^SQSTM1^ with that of α-tubulin, as described in the Materials and Methods Section. Symbols indicate significance, * *p* < 0.05, ** *p* < 0.01 CEN + IR vs. IR or Veh (Student’s *t* test). Panel (**e**): SA-β-GAL staining of SAOS400 cells irradiated (5 Gy) and then treated for 144 h with 200 μg/mL of CEN or their combination Symbols indicate significance + *p* < 0.05 IR or CEN vs Ctrl *** *p* < 0.01 CEN + IR vs. IR and CEN. Panel (**f**): Micrographs of representative fields of SAOS400 cells after SA-β-GAL staining (200× magnification).

To verify whether irradiation and CEN induced apoptosis in SAOS400, we measured caspase-3 activity, a reliable marker for cells that are dying, or have died by apoptosis. The negative results of the caspase-3 enzymatic assay, shown in Figure 5a after IR, CEN, and combined treatments, stimulated the investigation of other cellular phenotypes involved in this process. Using autophagic and senescence markers, we evaluated the effect of γ-rays on SAOS400 cells in combination with CEN. Figure 5b shows that after 120 h of treatment, both IR and CEN were able to significantly increase the number of autophagosomes. However, when SAOS400 cells were irradiated and subsequently treated with CEN, the cytoplasmic autophagosomes increased further (>55%) with respect to Veh. The increase was also significantly different compared to single treatments. This trend was confirmed by measuring the autophagic markers shown in Figure 5c,d. Following CEN treatment, Beclin-1 expression was 2.5 times higher than Veh, and an approximately 2-fold increase was detected in the combined treatment, IR plus CEN. In contrast, the expression of Beclin-1 decreased after irradiation and the same occurred for p62^SQSTM1^, indicating that IR modulated autophagic flux differently than CEN in SAOS400 cells.

In Figure 5c it is shown that the expression level of p16^INK4^ decreased (about 20%) after treatment with γ-rays or CEN, and this decrease was even more relevant (<50%) in the combined treatment, a trend confirmed by SA-β-GAL staining, as shown in Figure 5e,f. These results suggest that both CEN and γ-rays produce a senomorphic (for the attenuation of the senescent phenotype) effect or a reduction of senescent cells interfering with cell cycle control. The senolytic effect was evident when SAOS400 cells were treated with radiation plus CEN, stimulating cell death (senolysis).

### 2.6. CEN and γ-Rays Synergistically Induce Cell Death in the SAOS400 Cell Line

**Figure 6:**
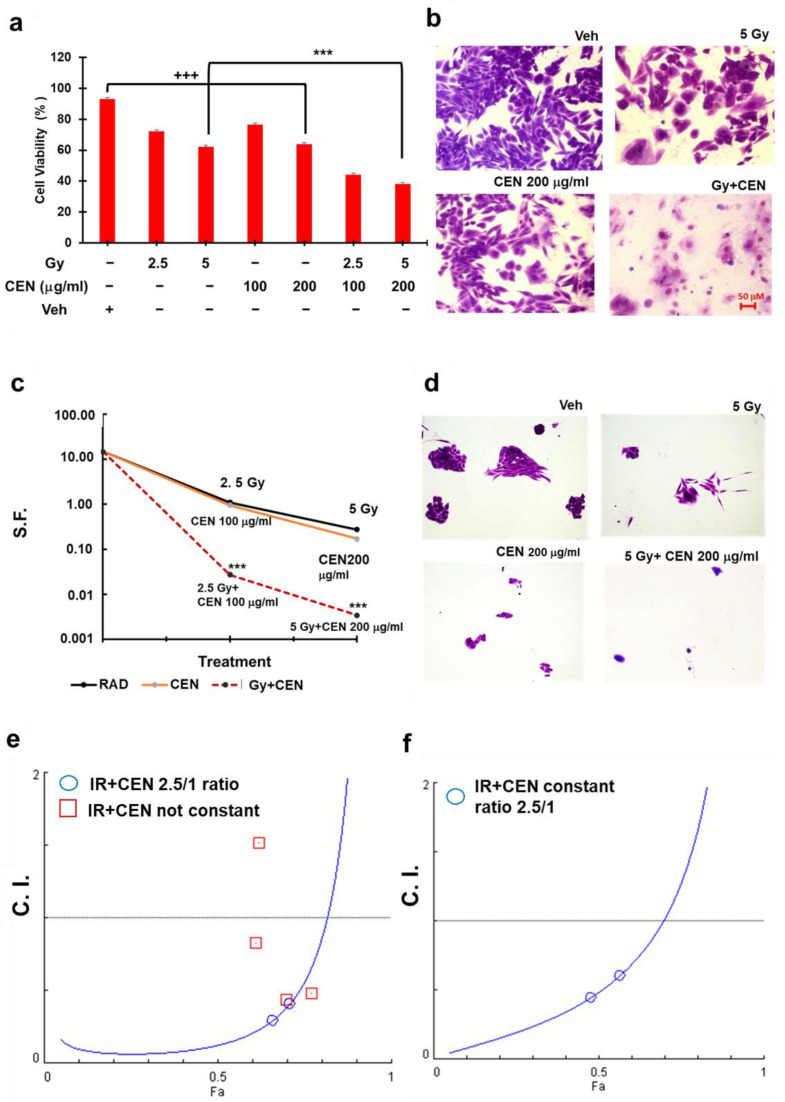
Irradiation and CEN synergistically induce cell death in the SAOS400 cell line. Panel (**a**): Crystal Violet assay in SAOS400 irradiated with 2.5 or 5 Gy and treated for 120 h with 100 or 200 μg/mL of CEN or their combination. The bar graphs represent the mean ± SD, and symbols and the linear bar indicate significance: +++ *p* < 0.001 Veh vs. IR- or CEN-treated cells, *** *p* < 0.001 CEN + IR vs. single treatments, Student’s *t* test. (**b**) Micrographs (200× magnification) of representative fields of SAOS400 cells treated as described in (**a**) and subsequently fixed and stained with Crystal Violet. Panel (**c**): Clonogenic assay. SAOS400 cells were irradiated with 2.5 or 5 Gy or incubated with 100–200 μg/mL of CEN and their combination for 11 days. Bar graphs represent the mean of the surviving fraction (SF) ± SD; in other words, the colonies that survived after each treatment (CEN, radiation, or combinations) with respect to untreated cells considering the plating efficiency in the clonogenic assay, as explained in the Materials and Methods Section. Symbols indicate significance: *** *p* < 0.001 CEN + IR vs. single treatments, Student’s *t* test. Panel (**d**): Micrographs (100× magnification) of representative clones of SAOS400 cells treated as described in panel (**c**) and subsequently fixed and stained with Crystal Violet**.** Panel (**e**): Isobologram that reproduces the calculation of the combination index (C.I., blue circle). The affected fractions (fa) were calculated based on the results obtained from the Crystal Violet assay using constant concentrations (radiation/CEN dose ratio 2.5/1). Panel (**f**): Isobologram showing the results of the C.I. analysis, where fa were calculated from the results of the clonogenic assay using constant (radiation/CEN dose ratio 2.5/1) (blue circles) or not constant concentrations (red squares).

To verify whether simultaneous administration of radiation and CEN could induce cell death, we pretreated SAOS400 cells with two different doses of γ-rays (2.5 and 5 Gy) followed by two doses of CEN (100–200 μg/mL) and their combination for 120 h (Figure 6a,b). Even if both CEN (Figure 2) or γ-rays showed a cytostatic effect on SAOS400 cells, only the combined treatment of CEN plus γ-rays caused a massive and significant cell death of approximately 55–60% (Figure 6a,b). To evaluate the proliferative response of SAOS400 cells to CEN after prolonged exposure (11 days), we used a clonogenic assay, repeating the same treatment and protocol described above (Figure 6c). These experiments allowed us to calculate the affected fraction (fa, that is, the percentage of dead cells, for each experimental point) and calculate the combination index (C.I., Figure 6e,f). The values reported in the isobolograms in Figure 6e,f have also been summarized in Table 1 for both cell viability and clonogenic assays. Briefly, Table 1 presents the results of complex data obtained by Combosyn software 1.0 after immission of the experimental values relative to the dose of drugs (in our article, CEN 100 or 200 μg/mL *w*/*v*) or radiation (in our paper, γ-rays doses expressed as 250 rad corresponding to 2.5 Gy or 500 rad corresponding to 5 Gy) and the effect expressed as percentage of dead cells (fa) after single treatments or combined treatments in a constant ratio of CEN and radiation doses (2.5/1 = 250 rad/100 μg/mL CEN; 500 rad/200 μγ/mL CEN). Since the C.I. was <1, we concluded that the combined treatment of IR plus CEN was highly synergistic under all experimental conditions tested.

### 2.7. CEN and γ-Rays Induce Lethal Autophagy in the SAOS400 Cell Line

**Figure 7:**
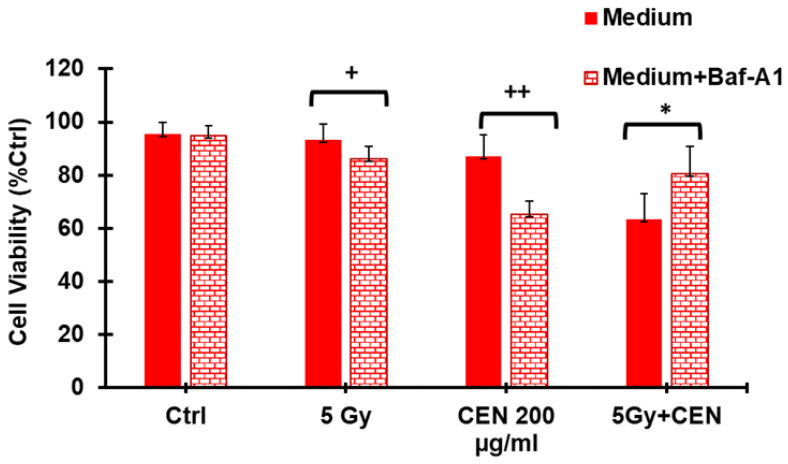
IR and CEN induce protective autophagy in the SAOS400 cell line, but their combination results in lethal autophagy (ADCD). SAOS400 cells were treated with γ-rays (5 Gy), CEN (200 μg/mL), or their combination for 72 h in the absence or presence of the autophagy inhibitor Baf-A1 (10 nM). At the end of incubation, a Crystal Violet assay was performed to calculate cell viability under the different experimental conditions. The bar graphs represent the mean ± SD, and symbols and the linear bar indicate significance: + *p* < 0.05 vs. IR + Baf-A1, ++ *p* < 0.01 CEN vs. Baf-A1, * *p* < 0.05 IR + CEN vs. IR+CEN+Baf-A1, Student’s *t* test.

We observed that the cell death seen in SAOS400 could not be attributed to apoptosis since no caspase activation was measured in the combined treatment of IR plus CEN (Figure 5a). Therefore, we used Baf-A1, an agent that inhibits autophagosome–lysosome fusion, to verify the type of autophagy induced by CEN, γ-rays, and their combination. We found that both IR and CEN induced a protective form of autophagy in SAOS400 cells because when Baf-A1 was present in the cell culture medium, a slight but significant decrease in cell viability was observed after IR (<10%) and CEN (<20%) mono treatments (Figure 7). However, in the combined treatment, CEN plus IR, a lethal form of autophagy (or ADCD) was evident since the presence of Baf-A1 reduced cell death and, consequently, increased cell viability (>20%). This phenomenon, known as the ‘autophagic switch’, may be partly responsible for the massive cell death observed in Figure 6.

### 2.8. CEN and γ-Rays Reduce Intracellular ATP and Activate AMPKα in the SAOS400 Cell Line

**Figure 8:**
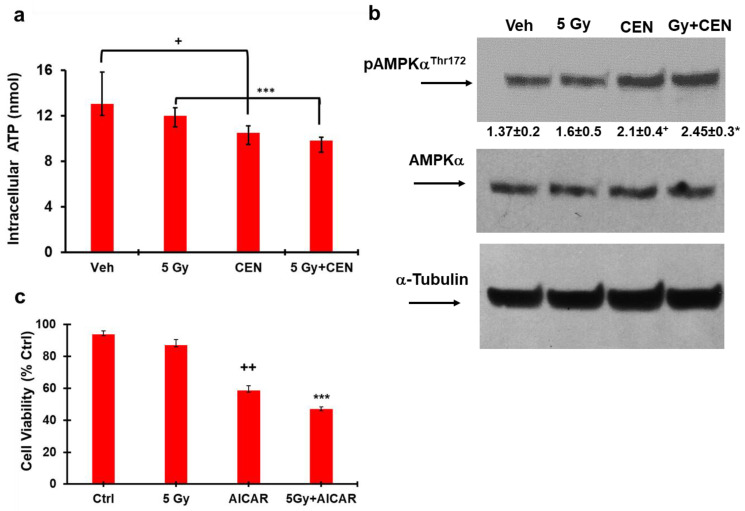
CEN-induced autophagy in SAOS400 cells was related to intracellular ATP levels and AMPK activation. Panel (**a**): SAOS400 cells were treated for 24 h with vehicle (Veh), CEN, 5 Gy, or a combination of 5 Gy plus CEN. Subsequently, the intracellular concentration (nmol) of ATP was measured with a luminometer as described in the Materials and Methods Section. The bar graphs indicate the mean of three experiments (±SD) in quadruplicate. Symbols: + *p* < 0.05 Veh vs. CEN, *** *p* < 0.001 5 Gy and CEN vs. 5 Gy, Student’s *t* test. Panel (**b**): Immunoblot analysis for the expression of the active form of AMPK (pAMPKα^Thr172^). Cells were irradiated with 5 Gy and then treated with 200 μg/mL of CEN or their combination for 24 h. Values between panels (mean of two experiments with statistical significance: + *p* < 0.05 Veh vs. CEN, * *p* < 0.05 CEN vs. 5 Gy, Student’s *t* test) indicate a densitometric analysis of the expression, as described in the Materials and Methods Section. Panel (**c**): SAOS400 cells were irradiated (5 Gy) or incubated with the AMPK activator, AICAR (5 μM), or their combination. Cell viability was evaluated using Cy-Quant dye after 120 h of treatment. The bar graphs represent the mean ± SD, and symbols and the linear bars indicate significance: ++ *p* < 0.01 Ctrl vs. 5 Gy or AICAR, *** *p* < 0.001 5 Gy and AICAR single treatments vs. combined treatment AICAR + 5 Gy (Student’s *t* test).

Previous data obtained in SAOS cells showed that CEN-induced autophagy was related to a decrease in intracellular levels of ATP and a concomitant increase in AMPK activity [7]. We verified whether the AMPK pathway was also involved in the SAOS400 response to IR, CEN, and CEN plus IR. Figure 8a shows that IR does not modify intracellular ATP levels; on the contrary, CEN and CEN plus IR were able to reduce intracellular ATP levels by about 20% after 24 h and, at the same time, CEN upregulated the activity of AMPK, as demonstrated by the 65% increase in the expression of its phosphorylated form, pAMPK Thr172, with respect to untreated cells (Figure 8b). The densitometric analysis in Figure 8b also shows that the expression of pAMPKα^Thr172^ was not affected by IR (5 Gy); in fact, the combined treatment did not significantly increase AMPK activity over CEN monotreatment. To confirm the involvement of AMPK in the sensitization of SAOS400 cells to IR treatment, we used a cell permeable activator, AICAR (Acadesine/AICA Riboside), which mimics the effects of AMP on the catalytic site of the enzyme [26]. Figure 8c shows a significant decrease of about 35% in cell viability after SAOS400 cell incubation in the presence of AICAR, in agreement with the result of CEN treatment (Figure 6a). The combination of AICAR plus IR further reduced cell viability with respect to IR monotreatment, mirroring the effects of CEN and CEN plus IR on intracellular ATP and cell viability.

### 2.9. Role of β-Carotene in Cell Death Induced by CEN and γ-Rays in the SAOS400 Cell Line

**Figure 9:**
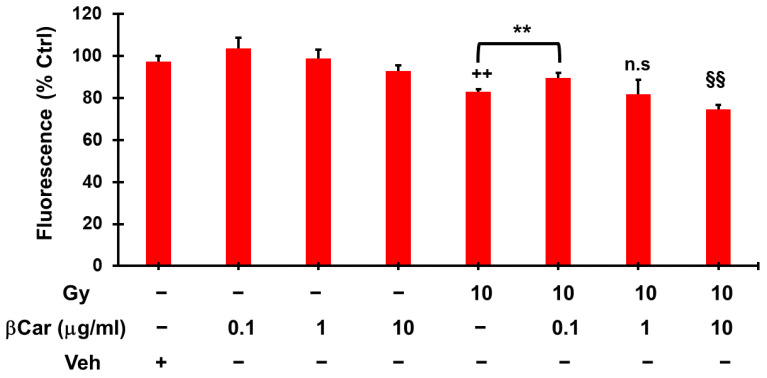
Cell death induced by CEN and radiation in SAOS400 is not dependent on β-carotene. SAOS400 cells were irradiated (10 Gy) and incubated for 144 h with β-carotene conveyed in cell culture medium by nanoemulsions with the indicated concentrations and respective combinations. Here, 1 μg/mL is approximately the dose of β-carotene present in 200 μg/mL of CEN. Cell viability was evaluated with the Cy-Quant fluorometric assay. The bar graphs represent the mean ± SD, and symbols and the linear bar indicate significance: ++ *p* < 0.01 10 Gy vs. Veh, ** *p* < 0.01 10 Gy vs. 0.1 μg/mL β-carotene, 10 Gy vs. 1 μg/mL β-carotene, n.s. not significant, §§ *p* < 0.01 10 Gy vs. 10 μg/mL β-carotene, Student’s *t* test.

The composition of the CEN extract in terms of carotenoids, vitamin E, and fatty acids is known [27] and includes the presence of β-carotene, one of the most representative biologically active carotenoids [1]. Therefore, we verified whether the effects of CEN on SAOS400 cells described in the present work could be completely or partially attributable to β-carotene. Figure 9 illustrates the results obtained with a Cy-Quant viability assay in cells treated for 144 h with a different range of β-carotene concentrations (0.1, 1, and 10 μg/mL), γ-rays (10 Gy), and their respective combinations. None of the doses of β-carotene, conveyed to cell culture medium with nanoemulsions, reduced cell viability in SAOS400 cells. The same is true for the association with γ-rays. The dose of 1 μg/mL of β-carotene, comparable to its concentration in 200 μg/mL of CEN, was unable to mimic the strong synergic effect of CEN plus IR, and only using a 10-fold higher dose of β-carotene (10 μg/mL) was there a slight reduction in cell viability (<8%) with respect to irradiated cells. Interestingly, the lower dose of β-carotene used in this experiment (0.1 μg/mL) seemed to have opposite effects, protecting cells against radiation-induced cell death.

## 3. Discussion

Treatment of therapy-resistant tumors remains a challenge. Deciphering the orchestration of signaling pathways in regulating cell death resistance will provide new insights into more effective therapeutic strategies. Recent research in cancer biology is based on the exploration of the underpinning mechanisms responsible for resistance to therapy in cancer cells and how drugs or radiation therapy in association with natural molecules present in food or a particular diet could pass this resistance, the so-called “precision nutrition” [28].

We previously reported that a carotenoid-enriched extract, obtained by supercritical extraction of CO_2_ (SC-CO_2_) from *Cucurbita moschata* sp., exerted an antiproliferative effect on different malignant cell lines (SAOS, Caco-2, and HG3) by activating a nonprotective form of autophagy [7,19]. Here, we demonstrate that the same extract reversed γ-ray resistance in the osteosarcoma-derived cell line SAOS400.

We compared the parental cell line SAOS with the derived counterpart, SAOS400, and found that the basal level of autophagosome and LC3-II was higher in radioresistant cells (Figure 1). This is in line with the concept that, similar to senescence, autophagy is a protective phenomenon in cancer cells, especially when they are subjected to stress conditions induced by chemotherapy or radiation therapy [29].

Interestingly, we found that both IR and CEN induce a protective cytostatic form of autophagy in radioresistant cells (Figure 2). However, while CEN produced a strong upregulation of the autophagic protein Beclin-1, whose stability indirectly depends on AMPK [26], the opposite occurred after irradiation (Figure 4). In other words, CEN acted on SAOS400 cells as a typical senotherapeutic molecule, since it reduced the percentage of senescent cells, as demonstrated by the lower levels of p16^INK4^ and SA-β-GAL staining [29]. This effect was linked to cell cycle arrest (Figure 2) without the sign of cell death (Figure 4 and Figure 5). In fact, neither radiation nor CEN was able to induce apoptosis (Figure 5a). However, when we combined γ-rays with CEN, a synergistic effect was measured in terms of cell death (Figure 6). This sensitization was not due to the activation of type I cell death or apoptosis, but rather to a lethal form of autophagy (Figure 7) and the concomitant elimination of senescent cells (Figure 5), also known as the senolytic effect.

The simultaneous presence of the two processes, e.g., senescence and autophagy, and the involvement of AMPK kinase are among the most promising and novel data emerging from this paper and deserve further investigation. One of the most potent inducers of autophagy is AMPK kinase, which plays a key role in cell energy homeostasis, primarily by activating glucose and fatty acid uptake and oxidation when cellular energy is low. Therefore, when a drop in intracellular ATP and a change in the ATP/AMP ratio are observed, the AMPK kinase is activated. Figure 8a showed that lower levels of intracellular ATP after CEN incubation were able to activate AMPK, and this activation was also present in the combined CEN plus IR, but not after IR monotreatment. For these reasons, we used the well-known AMPK activator, AICAR, to simulate the effects of CEN in our experimental system to mimic the effect of the “physiological modulator”, AMP. Figure 8c clearly shows that AICAR recapitulated the effects on cell viability obtained with CEN and CEN plus γ-rays.

AMPK activation after CEN incubation, but not after IR, can also explain the upregulation of Beclin-1. AMPK indirectly activates Beclin-1 throughout the phosphorylation of the Atg1/ULK1 initiation complex and the subsequent nucleation step that depends on the core complex Beclin1/Vps34/Vps15. Interestingly, a study by Song et al. also showed that AMPK-dependent phosphorylation is required at specific Beclin-1 sites, for example Ser90/93/96, to block the intracellular cysteine/glutamate antiporter system XC and induce ferroptosis through increased intracellular ROS, GSH depletion, and lipid peroxidation [30].

Beclin-1 can be seen as a molecular link between autophagy and apoptosis, since, when expressed, it associates through the BH3 domain with Bcl-2 or Bcl-X_L_, blocking the anti-apoptotic pathway regulated by these factors [31]. Although further studies are needed to confirm the importance of the AMPK pathway in the pro-autophagic and senolytic effects of CEN, our preliminary observations suggest that ATP/AMPK-dependent signaling may be central in the regulation of autophagy, apoptosis, and senescence in radioresistant cells (manuscript in preparation).

Recent works explore the interconnection between autophagy and apoptosis and the role of natural molecules, mainly polyphenols, in such a process. Shui et al. showed that isoquercitrin induced apoptosis and cytotoxic autophagy in cells of hepatocellular carcinoma through the AMPK/mTOR/p70S6K signaling pathway [32]. Another recent study demonstrated that quercetin, acting directly on PI_3_K and a new AKT inhibitor, STL-1, was able to induce both apoptosis and nonprotective autophagy in a chronic lymphocytic cell line [33]. These studies led to the hypothesis that natural molecules could act on intracellular signaling that converges on AMPK/AKT/mTOR to modulate autophagy, for example, by inducing an autophagic switch from a protective to a cytotoxic form. However, the exact mechanisms remain largely unknown.

Our data also showed that CEN acted differently in SAOS vs. SAOS400 cells, activating nonprotective autophagy in the former and a protective form in the latter. In this case, the “autophagic switch” was dependent on the cellular model and perhaps on the different nature of the inducer, the phytocomplex enriched in carotenoids.

According to the World Cancer Research Fund, there is strong evidence that high-dose supplementation of β-carotene is associated with an increasing risk of lung cancer, while for other types of cancer, the protective or harmful effects of carotenoids or carotenoid supplements containing foods require more detailed epidemiological and molecular studies. Analysis of the mechanistic evidence is complicated by the double nature of carotenoids, which are molecules that act as antioxidant or pro-oxidant compounds [1]. The data obtained with different concentrations of β-carotene, used as a representative molecule within the CEN composition, seem to confirm this assumption. In particular, we observed that a low concentration acted as a radioprotective molecule, while a higher concentration (>100-fold) slightly enhanced radiation toxicity in SAOS400 cells. Future evaluation of the intracellular redox status in the SAOS400 cell line after CEN treatment or the administration of different concentrations of β-carotene will clarify and, we hope, explain these paradoxical effects. Based on these results, we can exclude that β-carotene is the only molecule responsible for the biological effects observed with CEN or its association with IR. As an alternative explanation, we can evoke a synergistic contribution of all components present in the CEN phytocomplex as responsible for the observed biological effects. This possibility deserves further investigation.

**Figure 10:**
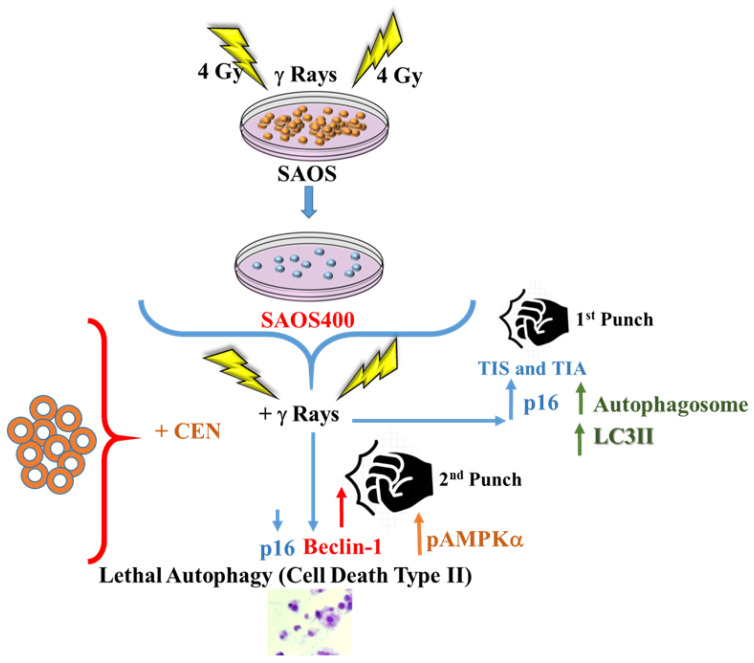
General scheme summarizing data obtained in the SAOS400 cell line after combined treatment with CEN and radiation. Therapy-induced autophagy and senescence can be seen as a double-edged sword in the context of radioresistance: They can represent a form of contrast to cell death, but also a ‘therapeutic vulnerability’ process. These processes can evolve in a new field of study that can be explored with both natural molecules and extracts enriched with natural bioactive molecules.

The final scheme presented in Figure 10 summarizes the results obtained in this work: therapy-induced autophagy and senescence (TIS and TIA) can act as a double-edged sword in the context of radioresistance. Both autophagy and senescence can be a form of resistance and contrast cell death in radioresistant cells, but also constitute a sort of therapeutic vulnerability. In this sense, senescent cells can be eliminated (senolysis) and protective autophagy switches to a nonprotective, or even lethal, process. In this scenario, the use of natural compounds, such as CEN, could represent a new tool to explore the mechanism of radioresistance. In fact, CEN clearly has an impact on some crucial pathways related to the senescence phenotype (p16^INK4^, SA-β-GAL) and autophagy activation (ATP/AMPK). However, even if neither CEN nor radiation alone are able to reach the threshold able to induce cell death in these cells, both treatments provide the first punch (proliferative arrest). The death threshold is reached and exceeded only when the cells receive the combined treatment that results in the second fatal punch (Figure 10).

## 4. Materials and Methods

### 4.1. Cell Culture and Irradiation

SAOS is a human osteogenic sarcoma cell line that expresses high levels of tissue-unspecific alkaline phosphatase activity, as described in [34]. It was donated by Prof. A. Oliva (L. Vanvitelli University, Naples, Italy), while SAOS400 were obtained from SAOS after irradiation with 4 Gy at time 0 and after 24 h, using a Gammacell Elite 1000 instrument (MDS Nordion, Ottawa, ON, Canada) equipped with a radioactive source that emits *γ*-rays (Cesium137 emitting approximately 2.5 Gy/min). After selection in vitro (about 4 weeks), the subpopulations enriched in radiation-resistant cells were named SAOS400 and used in the early passages. Both cells were cultured in high-glucose Dulbecco’s Modified Eagle Medium (DMEM, Euroclone, Milan, Italy) supplemented with 10% Fetal Bovine Serum (FBS) (Invitrogen Life Technologies (Milan, Italy)), 1% penicillin/streptomycin (Euroclone), 1% L-Glutamine, and 1% non-essential amino acids, in a humidified incubator at 37 °C with 5% CO_2_. The medium was changed every 48 h, and CEN or Veh was added again after the medium change. Two cell viability assays, Crystal Violet (Merck/Sigma, Milan, Italy) and Cy-Quant (Thermo-Fisher Scientific/Life Technologies Milan, Italy), were performed as reported [25,35]. The Crystal Violet assay is based on the staining of attached, fixed cells with a dye selective for proteins and DNA. Briefly, cells were fixed with 10% formalin for 15 min and 0.1% Crystal Violet (*w*/*v*) was added for 30 min. After washing, cells were solubilized with 10% acetic acid and absorbance was measured spectrophotometrically at 590 nm. Cy-Quant is a cell-permeant, fluorescent, DNA-binding dye (Cy-Quant nuclear stain) used in combination with a masking dye reagent (background suppressor) capable of quantifying cell proliferation and cytotoxicity. Briefly, a Cy-Quant mixture was added to the culture medium for 1 h at 37 °C and fluorescence was measured at the excitation wavelength of 485 and 530 nm emission in a multiplate reader (Synergy HT, BioTek, Milan, Italy). The two assays were performed simultaneously with cell counts (trypan blue) to confirm the cytotoxic and/or cytostatic effects induced by IR and/or CEN. Microphotographs of cells in representative fields after treatment were taken at 400× magnification using an invertoscope (Axiovert 200; Zeiss, Milan, Italy).

### 4.2. Preparation of Carotenoid Extract and Nanoemulsions

The extract was prepared from a typical product of the Campania region (Italy), the pumpkin variety “long Neapolitan pumpkin” or *Cucurbita moschata*, as described in a previous article [7]. Briefly, after grinding, the bioactive matrix (containing flesh and seeds 1/1, *w*/*w*) was extracted using the SC-CO_2_ technique (Super-Critical CO_2_). The oil obtained was characterized for the main lipophilic molecules (carotenoids, tocochromanols, and fatty acids), as reported in [27]. In particular, the total amount of carotenoids was 49.2 μg/100 g, with α-carotene and β-carotene representing 38.5% and 46.7% of the total, respectively. The enrichment in carotenoids made the preparation highly insoluble in aqueous solution. To favor bioavailability and preserve its stability in cell culture medium, the carotenoid preparation and β-carotene were conveyed with nanoemulsions.

Oil-in-water (o/w) nanoemulsions containing carotenoid-enriched extract (CEN) were prepared using a high-energy emulsification-evaporation technique [36]. CEN was prepared by mixing in a 1:3 ratio (*v*/*v*) with tetrahydrofuran (THF) containing 0.0025% (*v*/*w*) butylated hydroxytoluene (BHT) and adding it to an aqueous solution containing 0.3% Tween-80 organic/aqueous phase (volume ratio was 1:9). The emulsions were homogenized using an Ultra-Turrax homogenizer (T8, Ika-Werke, Staufen Germany) performing three cycles at 10,000× *g* for 2 min. To obtain nanoemulsions, the samples were sonicated (sonicator, XL Ultrasonic Processor, Misonix) as previously described [7]. After the solvent phase of the nanoemulsions under nitrogen vapors, the samples were sterilized on a 0.2 μm membrane. Control nanoemulsions (Veh) were prepared by replacing CEN with the same quantity of THF/BHT 0.0025% *v*/*w* in the aqueous solution. The concentration of carotenoids incorporated into the nanoemulsions was determined by extracting 0.5 mL of the nanoemulsions with hexane and absolute ethanol (2:1 *v*/*v*), as reported by Yuan et al. [37].

### 4.3. Colony Formation Assay

The colony formation assay was performed as described by Franken et al. [38]. SAOS400 cells were seeded at low density (1 × 10^3^ cells/well) in a 35 mm well plate, irradiated with the indicated doses of CEN and IR, and cultured at 37 °C for 11 days. This incubation time was carefully selected to allow an accurate count of colonies and to avoid clones overlapping. Cells were washed in phosphate-buffered saline (PBS; Euroclone), fixed with 10% formaldehyde in PBS, and stained with 0.5% Crystal Violet. The results were expressed as fractions of the number of clones with more than 50 cells compared to the untreated cells. The surviving fraction (SF) was calculated as follows: SF = colonies counted/(cells seeded × P.E./100), where P.E. represents the plating efficiency, defined as the percentage of seeded cells that formed colonies (comprising >50 cells) under specific culture conditions.

### 4.4. Cell Cycle Analysis

SAOS400 cells (1.5 × 10^6^/mL) were treated for the indicated times with CEN, harvested, counted with Trypan blue solution, fixed within ice-cold 70% ethanol, washed in PBS, and stained with 50 μg/mL of propidium iodide in the presence of 100 μg/mL of DNAse-free RNAase A for 1 h at 37 °C in the dark. After the staining procedure, cells were analyzed by flow cytometry (Facscalibur; BD Biosciences, Sparks, MD, USA) and DNA content was quantified using ModFit LT software 3.0 (Verity Software House, Inc., Topsham, ME, USA) to measure the percentage of diploid cell distribution in the different phases of the cell cycle [39].

### 4.5. Evaluation of Senescence, Apoptosis, and Autophagy Markers

To evaluate senescence markers, we used classical lysosomal SA-β-GAL staining. Cells (in the early passages) were seeded at sub-confluent density (1.0–1.5 × 10^4^/well) in 35 mm tissue culture plates. After 144 h of IR, cells were washed with PBS and fixed in 3% formaldehyde/PBS (Merck/Sigma) for 5 min at room temperature. Cells were then washed with PBS and stained in fresh X-Gal staining solution containing 1 mg/mL of 5-bromo-chloro-indolyl β-D galactoside (X-Gal; Merck/Sigma) in a buffer consisting of 40 mM of sodium phosphate, 150 mM of NaCl, 5 mM of C₆FeK₄N₆, and 2 mM of MgCl_2_, pH 6. After overnight incubation at 37 °C, cells were washed with PBS and microphotographs were taken with an invertoscope (Aziovert 200, Zeiss) [40]. Cells were visually scored as positive or negative for β-GAL staining. At least 100–200 cells were counted/sampled in duplicate and expressed as percentage of β-GAL-positive cells/total cells.

To determine caspase-3 enzymatic activity, SAOS400 cells were treated as indicated in the legend of Figure 5a for 24 h. Subsequently, cells were collected and centrifuged at 400× *g* for 5 min, washed twice in PBS, and suspended in lysis buffer (10 mM Hepes, pH 7.4, 2 mM ethylenediaminetetraacetic acid, 0.1% [3-(3-cholamidopropyl) dimethylammonio]-1-propanesulfonate, 5 mM dithiothreitol, 1 mM phenylmethylsulfonylfluoride, 10 μg/mL pepstatin-A, 10 μg/mL apronitin, 20 μg/mL leupeptin). Cell extracts (10 μg) were added to the reaction buffer and the conjugated amino-4-trifluoromethyl coumarin substrate (AFC): benzyloxycarbonyl-Asp (OMe)-Glu (OMe)-Val-Asp (OMe)-AFC (Z-DEVD-AFC) (ENZO Life Science, Milan, Italy). The samples were incubated at 37 °C for 30 min. Upon proteolytic cleavage of the substrate by caspase-3, the free fluorochrome AFC was detected by a multiplate reader (Synergy HT BioTek) with excitation at 400 ± 20 nm and emission at 530 ± 20 nm. To quantify the enzymatic activity, an AFC standard curve was assessed. Caspase-3-specific activity was calculated as nmol of AFC produced per min per μg proteins at 37 °C in the presence of a concentration of the saturating substrate (50 μM).

Autophagy was monitored using the Cyto-ID Autophagy Detection Kit that directly quantifies intracellular autophagosome (ENZO Life Science, Milan, Italy), as described in [7,41]. After incubation, cells were washed and incubated with the autophagy detection marker (Cyto-ID) and nuclear dye (Hoechst 33342). Cells were rinsed with assay buffer and photographed using a fluorescence microscope (Zeiss Axiovert 200). Finally, autophagosomes were quantified by normalizing green (Cyto-ID) and blue (Hoechst) fluorescence using a microplate fluorescence reader (Synergy HT BioTek). SAOS400 cells were incubated for the indicated times with IR, CEN (200 μg/mL (*w*/*v*), 20 μM of CQ, and 2 μM of ATRA (positive controls). In particular, we estimated the impact of autophagy (protective vs. not protective or cytotoxic) on cell viability after IR. The AICAR (Acadesine/AICA Riboside), protein kinase (AMPK) activator was purchased from Merck/Millipore.

### 4.6. Immunoblotting

Cells were lysed using a lysis buffer containing protease and phosphatase inhibitors, as reported in [25]. After protein concentration [42], the total lysates (20 μg/lane) were loaded onto a 4–12% precast gel (Novex Bis-Tris precast gel; Thermo-Fisher Scientific/Life Technologies) using 50 mM of MES (2-(Nmorpholino) ethanesulfonic acid) buffer at pH 7. Immunoblots were performed adopting standard procedures using polyvinylidene fluoride (PVDF) membranes incubated for about 16 h with the following primary antibodies: anti-p16^INK4^ (cat. # SC-759), anti-LC3I-II (cat. # 12741S), anti-p62^SQSTM1^ (cat. # 5114S), anti-Beclin-1 (cat. # 4122S), anti-pAMPKα^Thr172^ (cat. # 2535S), and anti-AMPKα (cat. # 5832S), from Cell Signaling (diluted 1:1000 in Tween-20 Tris buffer salinum, T-TBS, containing 3% bovine serum albumin), and anti-α-tubulin from Merck/Sigma (Cat. # T9026) diluted 1:3000 with 3% bovine serum albumin in T-TBS. The membranes were washed in T-TBS and finally incubated for 2 h with a horseradish peroxidase-linked secondary antibody (diluted 1:20,000 in T-TBS) raised in mouse or rabbit. Immunoblots were developed using the ECL Prime Western blotting detection system kit (GE Healthcare, Milan, Italy). Band intensities were quantified as optical density on a Gel Doc 2000 apparatus (Bio-Rad Laboratories, Milan, Italy) and Multianalyst software version 1.1 (Bio-Rad Laboratories).

### 4.7. Intracellular ATP Levels

To measure intracellular ATP levels, we used the ViaLight plus kit (Lonza; Euroclone, Milan, Italy) based on the bioluminescence measurement of ATP in metabolically active cells. According to the manufacturer’s instructions, SAOS400 cells (2500/well) were seeded in a 96-well tissue culture plate. At the end of incubation in the presence of γ-rays, CEN (200 μg/mL, *w*/*v*), or IR plus CEN (Veh was used as control), the medium was removed, and cells were incubated with 50 μL of lysis buffer for 10 min at room temperature. Subsequently, the reaction buffer was added, and the samples were incubated for 2 min at room temperature. Finally, the plate was read on a microplate luminometer (Synergy HT) and the results were expressed as nanomoles of ATP after extrapolation of luminescence values using a linear calibration curve made using standard ATP, as described in [7].

### 4.8. Evaluation of the Combination Index

The combination index (CI) was calculated according to the mathematical model of Chou and Talalay for drug interactions, as previously reported [18,43,44]. Dose-response curves were based on the affected fraction (fa), indicating the percentage of dead cells compared to those of the untreated cells. Dose effect analysis and CI for the combined treatment groups of CEN plus IR at the indicated doses (in the figures, these data are expressed in isobolograms) were generated using Compusyn software 1.0, freely available at www.combosyn.com (accessed on 3 February 2022). Compusyn software 1.0 is based on the median effect equation (MEE) and calculates several data points. The dynamics of interactions of multiple entities resulted in the general combination index equation (CIE), algorithm, and computer simulation, which quantitatively determine synergism (CI < 1), additive effect (CI = 1), and antagonism (CI > 1), automatically.

### 4.9. Statistical Analysis

The results have been expressed as mean ± standard deviation (SD) based on the values obtained from independent experiments carried out in triplicate or quadruplicate. Differences between two groups were analyzed using Student’s *t* test (Excel^MS^ software 2016) and significance was established at *p* < 0.05, with specific values indicated in the legends of the figures.

## 5. Conclusions

The present work investigated the ability of carotenoid-enriched nanoemulsions (CEN) to sensitize the radioresistant osteosarcoma cells to cell death. We demonstrated that in SAOS400 cells, CEN and γ-rays synergistically eliminated senescent cells (senolysis) and activated a lethal form of autophagy, which was dependent upon AMPK activation and a decrease in intracellular ATP. For the first time, to our knowledge, data have been presented on the senolytic activity of carotenoids, a class of phytochemicals challenging to study because of their low solubility and stability. To reach this goal, the present work took advantage of innovative methods based on nanoemulsions employed as a carrier to deliver the mixture of carotenoids to radioresistant cells. Finally, we excluded the possibility that β-carotene, abundantly present in CEN, was responsible for reversing the resistance of SAOS400 cells to radiation. We can conclude that several bioactive compounds, beyond β-carotene, are present in CEN and act synergistically to sensitize radioresistant cancer cells to cell death, stimulating further investigations in animal models.

## Figures and Tables

**Table 1:** Calculation of C.I. in the combined treatment of CEN plus IR.

**Cristal Violet Assay**			
**CEN (μg/mL)**	**IR (Gy)**	**Effect ^§^**	C.I. *
100	2.5	0.476	0.44
200	5	0.565	0.60
**Clonogenic Assay**			
**CEN (μg/mL)**	**IR (Gy)**	**Effect ^§^**	C.I. *
100	2.5	0.66	0.29
200	5	0.70	0.40
100	5	0.61	0.82
200	10	0.77	0.48
200	5	0.70	0.43

^§^ Effects and fa values were obtained with the Crystal Violet assay or the clonogenic assay. * C.I. values were calculated using Compusyn software 1.0, as described in the Materials and Methods Section, at constant (radiation/CEN dose ratio 2.5/1 blue values) or not constant (red values) doses of radiation and CEN.
